# The Roles of the Cortical Motor Areas in Sequential Movements

**DOI:** 10.3389/fnbeh.2021.640659

**Published:** 2021-06-09

**Authors:** Machiko Ohbayashi

**Affiliations:** ^1^Department of Neurobiology, University of Pittsburgh School of Medicine, Pittsburgh, PA, United States; ^2^Systems Neuroscience Center, Center for the Neural Basis of Cognition, University of Pittsburgh, Pittsburgh, PA, United States

**Keywords:** sequential movements, motor skills, learning, motor cortex, premotor cortex, SMA

## Abstract

The ability to learn and perform a sequence of movements is a key component of voluntary motor behavior. During the learning of sequential movements, individuals go through distinct stages of performance improvement. For instance, sequential movements are initially learned relatively fast and later learned more slowly. Over multiple sessions of repetitive practice, performance of the sequential movements can be further improved to the expert level and maintained as a motor skill. How the brain binds elementary movements together into a meaningful action has been a topic of much interest. Studies in human and non-human primates have shown that a brain-wide distributed network is active during the learning and performance of skilled sequential movements. The current challenge is to identify a unique contribution of each area to the complex process of learning and maintenance of skilled sequential movements. Here, I bring together the recent progress in the field to discuss the distinct roles of cortical motor areas in this process.

## Introduction

The production of sequential movements is a fundamental aspect of voluntary behavior. Many of our daily actions, such as playing a musical instrument, handwriting, typing, etc., depend on attaining a high level of skill in the performance of sequential movements. The performance of sequential movements can be acquired and improved to the expert level through extensive practice (Rosenbaum, 2010). Such performance can be maintained as a motor skill. How the brain binds elementary movements together into skilled sequential movements has been a fundamental problem of systems neuroscience.

The neural basis of sequential movements has been extensively studied in human and non-human primates. Human imaging studies have shown that a brain-wide distributed network, which is composed of the presupplementary motor area (pre-SMA), supplementary motor area (SMA), dorsal premotor cortex (PMd), primary motor cortex (M1), primary somatosensory cortex, superior parietal lobule, thalamus, basal ganglia, and the cerebellum, subserves the learning and performance of skilled sequential movements (e.g., Shibasaki et al., 1993; Grafton et al., 1994, 1995; Karni et al., 1995; Hikosaka et al., 1996, 2002; Sakai et al., 1998; Ungerleider et al., 2002; Dayan and Cohen, 2011; Hardwick et al., 2013). Studies in non-human primates showed that neural activity of these areas reflected aspects of sequences (e.g., Mushiake et al., 1991; Mushiake and Strick, 1993, 1995; Tanji and Shima, 1994, 1996b; Clower and Alexander, 1998; Nakamura et al., 1998; Miyachi et al., 2002; Lee and Quessy, 2003; Matsuzaka et al., 2007; Picard et al., 2013; Ohbayashi et al., 2016). Nevertheless, the results sometimes appear contradictory. This could be due to the various cognitive demands associated with different formulations of sequential tasks. Several features of sequential movement tasks can be identified, such as guidance (i.e., guided by external *vs*. internal cues), memory (i.e., guided by short-term *vs.* long-term memory), movement outcome (i.e., configuration *vs.* spatial position), and movement flow (i.e., with temporal separation *vs.* continuous). It was suggested that spatial and non-spatial sequences may be learned and controlled by different cortical circuits (Tanji, 2001; Ohbayashi et al., 2016). Furthermore, neural activity has been shown to change as a result of practice on motor skill tasks (e.g., Grafton et al., 1994; Karni et al., 1995; Sakai et al., 1998; Coynel et al., 2010).

In this review, I will focus on the roles of the SMA, PMd, and M1 in skilled sequential movements, i.e., those acquired through repetitive practice and internally generated from long-term memory. I will especially focus on the spatial sequence tasks as this type of task was used in non-human primate studies after extensive practice (Table 1). The current challenge is to identify a unique contribution of each area to the complex process of acquisition and retention of sequential movements. Interventional studies in non-human primates could represent a valuable complement to neuroimaging studies. These methods can critically address the causal relationship between the activity in a brain area and behavior. I will aim to integrate recent discoveries regarding the cortical control of skilled sequential movements at multiple levels of complexity by highlighting interventional (e.g., inactivation) studies in non-human primates.

**TABLE 1:** Non-human primate studies on memory-guided sequential reaching tasks.

Study	Area	Training duration	Method	Main findings
Lee and Quessy, 2003	SMA	1 day	Neural recording	About one-third of the neurons in the SMA displayed gradual changes in their activity across different trials when a particular movement sequence was repeatedly performed.
Mushiake et al., 1991	SMA, PMd, M1	3–5 months	Neural recording	More than a half of the SMA neurons were preferentially active during memory-guided sequential reaching (55% and 65% during the pre-movement and movement periods). More than a half of the PM neurons were preferentially active in visually guided reaching (55% and 64% during the pre-movement and movement periods). M1 neurons showed similar activity regardless of whether it was guided by memory or visual stimulus.
Clower and Alexander, 1998	SMA	–	Neural recording	Neurons in the SMA and pre-SMA reflected the numerical order of the specific movement component of a sequence.
Nakamura et al., 1998	SMA	8 months–2 years	Neural recording	Neurons in the SMA and pre-SMA respond preferentially to a specific order of movements.
Nakamura et al., 1999	SMA	8 months–2 years	Muscimol injection	Inactivation of the SMA did not disrupt the learning or performance of the sequential reaching guided by memory.
Picard and Strick, 1997	SMA	∼39 months	2DG	2DG uptakes in the SMA and pre-SMA were relatively low in the remembered sequential reaching movements.
Picard and Strick, 2003	SMA	12–17 months	2DG	2DG uptakes in the SMA and pre-SMA were substantial in the visually guided reaching.
Ohbayashi et al., 2016	PMd	>50 days	Neural recording, muscimol injection	Injection of muscimol disrupted the performance of the memory-guided sequential reaching, but not the visually guided reaching. Forty-three percent of the neurons were differentially active during the memory-guided sequential reaching and visually guided reaching.
Lu and Ashe, 2005	M1	∼6 months	Neural recording, muscimol injection	Neurons exhibited anticipatory activity related to specific sequences. After muscimol injection, the number of errors in the sequential movements increased.
Ohbayashi, 2020	M1	>100 days	Anisomycin injection, muscimol injection	Anisomycin injection disrupted the performance of the memory-guided sequential reaching, but not the visually guided reaching. Muscimol injection disrupted the performance of both the memory-guided sequential reaching and visually guided reaching.
Matsuzaka et al., 2007	M1	>2 years	Neural recording	∼40% of the task-related neurons were differentially active during the memory-guided sequential reaching and visually guided reaching.
Picard et al., 2013	M1	∼1–6 years	2DG, neural recording	2DG uptake was lower in monkeys that performed sequential reaching guided by memory compared with the 2DG uptake in monkeys that performed visually guided reaching. 2DG uptake was lower in monkeys that were trained for a longer duration.

**SMA*, supplementary motor area; *PMd*, dorsal premotor cortex; *M1*, primary motor cortex; *2DG*, 2-deoxyglucose.–: Training duration was not provided in the manuscript.*

## Learning of Skilled Sequential Movements

**FIGURE 1:**
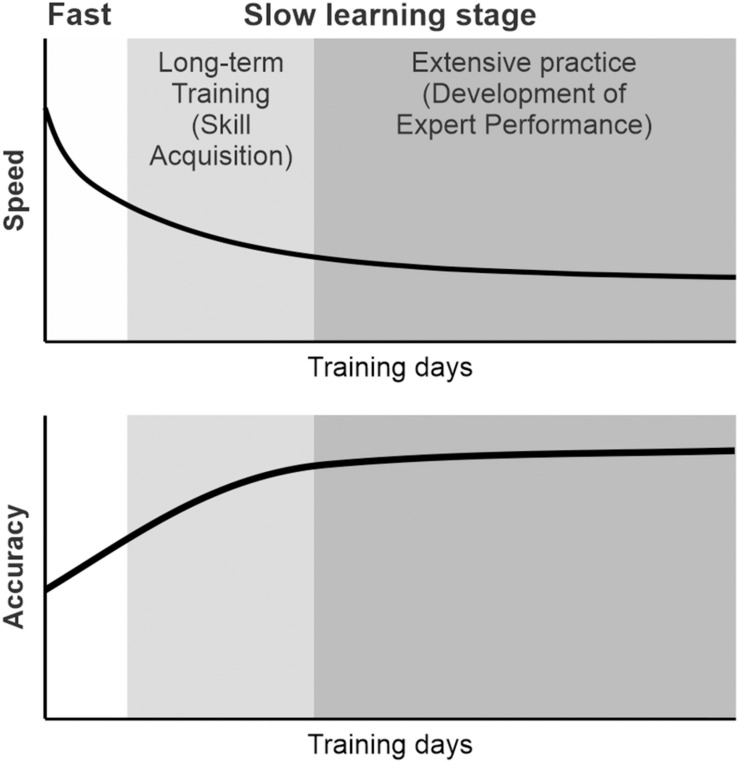
Schematic diagram of the learning of skilled sequential movements. During learning, individuals seem to go through several learning stages. The subject improved the performance of sequential movements relatively fast initially and, later, more slowly over multiple sessions of practice. Through repetitive practice, subjects improve their speed and accuracy of sequential movements. The performance of sequential movements can be improved to the expert level through extensive practice and can be maintained as a motor skill.

An important characteristic of learning skilled sequential movements is that individuals seem to go through several learning stages (Fitts and Posner, 1967; Hikosaka et al., 2002; Doyon et al., 2003; Doyon and Benali, 2005; Rosenbaum, 2010; Dayan and Cohen, 2011; Schmidt and Lee, 2011). An improvement of performance can be detected as changes in the speed and accuracy during learning (Figure 1). During an initial learning stage of skilled sequential movements, a subject improves performance of sequential movements relatively fast. The subject tends to make a large number of errors and highly variable movements with a lack of consistency from trial to trial, but achieves large performance improvements (Fitts and Posner, 1967; Rosenbaum, 2010; Dayan and Cohen, 2011; Schmidt and Lee, 2011). Later, the subject improves the performance more slowly over multiple sessions of practice by making fewer and smaller errors. The durations of the learning stages are highly specific to the tasks, subjects, and definitions. For example, the fast stage of learning to perform a sequential finger opposition task was defined as an initial within-session improvement phase in a human study (Karni et al., 1995, 1998), whereas the fast stage of learning to play an advanced piano piece may last months. Monkeys performing a sequential reaching task rapidly improved their response speed over about 50 days (Matsuzaka et al., 2007). Despite timing differences, the learning curves on different skill tasks follow the same patterns of initially fast, then slowing performance improvements with further practice. Through extensive and repetitive training, subjects can further improve their performance to the expert level. Then, the skill will become almost automatic with very small variability and small improvement (Fitts and Posner, 1967; Rosenbaum, 2010; Schmidt and Lee, 2011).

The progress in the learning of sequential movements is associated with a shift in functional MRI (fMRI) activation from the anterior regions to the posterior regions of the brain (Grafton et al., 1994; Sakai et al., 1998; Coynel et al., 2010). The change in fMRI activation is shown to be associated with improvement in the task performance during learning (Bassett et al., 2015; Reddy et al., 2018). This suggests that the extent of contribution of each area may change during learning. Hikosaka et al. (2002) proposed that a subject learns the spatial features of sequences during the fast learning stage and then learns the motor features of the sequences during the slow learning stage. In the following sections, I will discuss the contributions of the SMA, PMd, and M1 to the learning and performance of spatial sequence tasks and how the skilled sequential movements are maintained after extensive practice.

## Supplementary Motor Area

Classically, the preparation for and the generation of sequential movements have been thought to depend on the supplementary motor area (SMA) and the pre-SMA (Roland et al., 1980; Brinkman, 1984; Goldberg, 1985; Dick et al., 1986; Halsband, 1987; Halsband et al., 1993; Tanji and Shima, 1994, 1996a,b; Grafton et al., 1995; Shima et al., 1996; Tanji et al., 1996; Gerloff et al., 1997; Picard and Strick, 1997, 2001; Nakamura et al., 1998; Shima and Tanji, 1998, 2000; Tanji, 2001; Hikosaka et al., 2002). Human patients with lesions that include these areas had deficits in performing self-initiated movements, sequential movements, and/or speech (Goldberg, 1985; Dick et al., 1986; Halsband et al., 1993). In agreement with the reports of human patients, studies in non-human primates clearly demonstrated the contributions of the SMA and pre-SMA to the learning or performance of sequence tasks composed of non-spatial movements (Brinkman, 1984; Halsband, 1987; Tanji and Shima, 1994, 1996a,b; Shima et al., 1996; Tanji et al., 1996; Shima and Tanji, 1998, 2000, 2006; Tanji, 2001; Table 2). Neural recordings in monkeys demonstrated that neurons in the SMA and the pre-SMA respond preferentially to a specific order of movements rather than a single movement (Tanji and Shima, 1994, 1996a; Shima and Tanji, 2000). The inactivation of these areas in monkeys demonstrated the contributions of the SMA and pre-SMA in the performance of a sequence composed of non-spatial movements (Shima and Tanji, 1998). When the SMA or pre-SMA was bilaterally inactivated by injecting muscimol (GABA_*A*_ agonist), the inactivation disrupted the monkey’s performance of sequences of arm movements guided by memory, leaving the execution of simpler, single movements unaffected (Shima and Tanji, 1998).

**TABLE 2:** Non-human primate studies on non-spatial sequence tasks guided by memory.

Study	Area	Training duration	Method	Main findings
Tanji and Shima, 1994	SMA	–	Neural recording	A group of neurons exhibited activity related to a predetermined sequence of movements.
Shima et al., 1996	Pre-SMA	–	Neural recording	A group of pre-SMA cells were active when a monkey was required to switch a sequence of movements to perform the next one.
Shima and Tanji, 1998	SMA, pre-SMA	–	Muscimol injection	An inactivation of either the SMA or the pre-SMA disrupted the performance of memorized sequential movements.
Shima and Tanji, 2000	SMA, pre-SMA	–	Neural recording	Neurons in both the SMA and pre-SMA exhibited activity at different phases in the task.
Shima and Tanji, 2006	Pre-SMA	–	Neural recording	A group of pre-SMA neurons represented odd-numbered trials within the sequential movements; others represented even-numbered trials.

**pre-SMA*, presupplementary motor area; *SMA*, supplementary motor area.–: Training duration was not provided in the manuscript.*

On the other hand, non-human primate studies using spatial sequence tasks suggested that spatial and non-spatial sequences may be learned and controlled by different cortical circuits (Tanji, 2001; Ohbayashi et al., 2016). Even though neural activity reflected a specific order of movements in a sequence in both types of tasks, the results of an inactivation study using a spatial sequence task were different from the results using the non-spatial sequence task. Neurons in the SMA and the pre-SMA respond preferentially to a specific order of movements rather than a single movement during the performance of internally generated spatial sequence tasks (Clower and Alexander, 1998; Nakamura et al., 1998; Lee and Quessy, 2003). The activity of the SMA neurons reflected a particular serial position in a sequence (Lee and Quessy, 2003). The pre-SMA is particularly active for the learning of new sequences of movements, but not the production of movement components (e.g., reaching) (Hikosaka et al., 1996). Furthermore, the 2-deoxyglucose (2DG) signals of these areas after extensive practice (>12 months) reflected the effect of long-term training on spatial sequences (Picard and Strick, 1997, 2003). The 2DG signal is suggested to be associated with presynaptic activity at both excitatory and inhibitory synapses and reflects the metabolic activity of synapses (discussed in Picard and Strick, 2003). In the studies, the monkeys were trained on remembered sequential movements or visually guided reaching for years (>12 months). After extensive practice, both the SMA and the pre-SMA displayed substantial uptakes of 2DG in association with visually guided reaching movements (Picard and Strick, 2003). On the other hand, 2DG incorporation in the SMA and pre-SMA was relatively low in the case of remembered sequential reaching movements (Picard and Strick, 1997). The differential metabolic activities of the pre-SMA and SMA in the two tasks suggested that these areas may be reorganized with overtraining on the remembered sequences after extensive practice. Therefore, the results from neural recording and 2DG showed that neurons in both the SMA and pre-SMA may play roles in the learning and performance of spatial sequence tasks.

Nevertheless, an inactivation study using spatial sequences provided results different from the inactivation study using non-spatial sequences. Hikosaka’s group trained monkeys to learn a spatial sequence of reaching movements to targets (Nakamura et al., 1999). In their sequence task, reaching movements in space were required, so that the selection of spatial end points in sequential reaching was a critical factor to control. When the pre-SMA was bilaterally inactivated by injecting muscimol, the inactivation disrupted the monkeys’ learning of a new sequence of movements, but not the performance of the memorized sequence of movements. Interestingly, local inactivation of the SMA did not significantly disrupt the learning or the performance of the sequential reaching task (Nakamura et al., 1999). The result suggests that the SMA may not be critically involved in the performance of this type of spatial sequences at the tested stage of learning, even though neurons exhibited the sequence-related activity. Clearly, the inactivation results highlight the most unique contribution of the targeted area to the sequence task.

Taken these findings together, the pre-SMA seems to be more critically involved in the cognitive aspects of acquiring a novel sequence of movements compared to the SMA (Hikosaka et al., 1996; Shima et al., 1996; Nakamura et al., 1998, 1999; Shima and Tanji, 2000, 2006). The SMA seems to be involved in the temporal organization of multiple non-spatial movements into a sequence (Boecker et al., 1998; Shima and Tanji, 1998; Tanji, 2001; Nachev et al., 2008; Orban et al., 2010; Wiener et al., 2010; Cona and Semenza, 2017). On the other hand, for the spatial sequence tasks, even though neural activity of the SMA neurons reflected aspects of sequences, its role is still debatable and needs to be further investigated. The results of the spatial sequence task suggested that the effect of muscimol injection in the SMA could be compensated by another motor area, possibly the PMd, which is anatomically connected with both the SMA and M1. In the next section, I will discuss the role of the PMd in the performance of internally generated sequential movement tasks.

## Dorsal Premotor Cortex

The dorsal premotor cortex (PMd) has been regarded as the area for the visual guidance of motor behavior in many studies (Kalaska and Crammond, 1995; Johnson et al., 1996; Wise et al., 1997; Hoshi and Tanji, 2007; Averbeck et al., 2009). Moreover, the PMd is suggested to be involved in the cognitive aspects of visually guided motor tasks, such as mental rehearsal and a decision making (Cisek and Kalaska, 2002, 2004, 2005; Pesaran et al., 2008). Considerable evidence suggests that the PMd is specifically involved in the guidance of movements based on memorized arbitrary sensorimotor associations (Passingham, 1988; Mitz et al., 1991; Kurata and Hoffman, 1994). Firstly, lesions or the inactivation of the PMd produces deficits on tasks that rely on the associations between an arbitrary visual cue (e.g., color or shape of a visual stimulus) and a movement (Halsband and Passingham, 1982; Passingham, 1988; Kurata and Hoffman, 1994). For example, Kurata and Hoffman trained monkeys to learn the visuo-motor association task in which the monkeys were required to move their wrist to the right or the left direction based on the color of a conditional cue (Kurata and Hoffman, 1994). Then, they locally inactivated the PMd by injecting a small amount of muscimol at sites where the preparatory neural activity was recorded during the performance of the conditional visuo-motor association task. The local inactivation of PMd disrupted the monkeys’ performance of the visuo-motor association task. Secondly, neurons in the PMd show a sustained activity that is specifically related to the performance of these visuo-motor association tasks (Kurata and Wise, 1988; Mitz et al., 1991; Kurata and Hoffman, 1994). The PMd neurons showed sustained activity after the presentation of the arbitrary visual cue during the movement preparation period (Kurata and Wise, 1988; Kurata and Hoffman, 1994). Although these findings are in line with the proposal that the PMd plays a crucial role in the visual guidance of movements in general, they specifically point to the important contribution of the PMd to memory-guided movements in which selection, preparation, and execution of movements are guided by memorized visuo-motor associations (Halsband and Passingham, 1982; Wise, 1985; di Pellegrino and Wise, 1993; Kurata and Hoffman, 1994).

**FIGURE 2:**
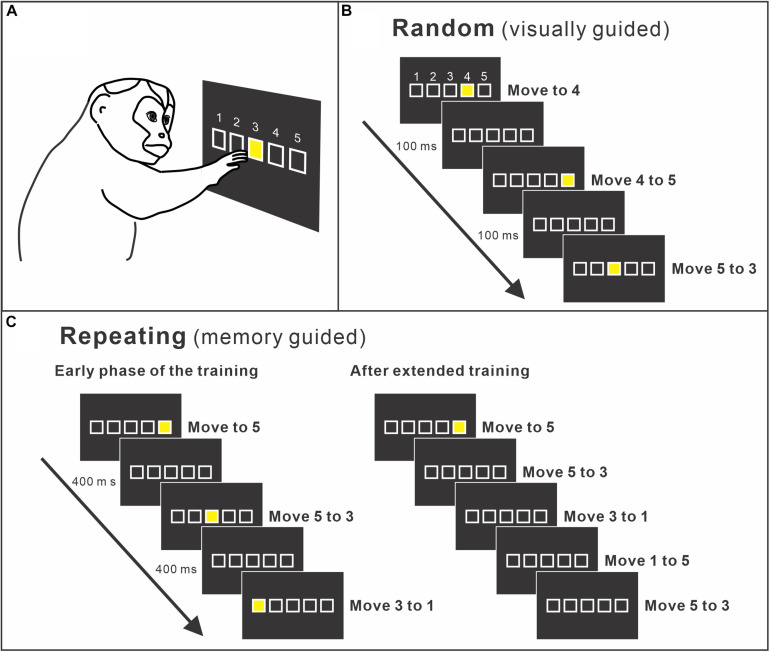
**(A)** Apparatus of the training setup. A monkey sits in front of a touch-sensitive monitor. To make a correct response, the monkey is required to contact a yellow target cue displayed on the touch monitor. The *yellow target* is presented at one of five squares displayed on the touch monitor. Squares are arranged in a horizontal row and identified as numbers *1* to *5* from *left* to *right*. **(B)** Random task. A new target cue is presented in pseudo-random order in one of the five squares. A new target is presented 100 ms after the monkey made a correct response or immediately after an error. Therefore, the monkey performs visually guided reaching from a target to the next target. **(C)** Repeating task. Targets are presented according to a predetermined sequence (*left*). As the monkey learns the sequence, the monkey started to touch the target in the sequence before the presentation of the visual cue (*right*). After extended practice, the monkeys perform the task without help of visual cues (modified from Ohbayashi and Picard, 2020).

**FIGURE 3:**
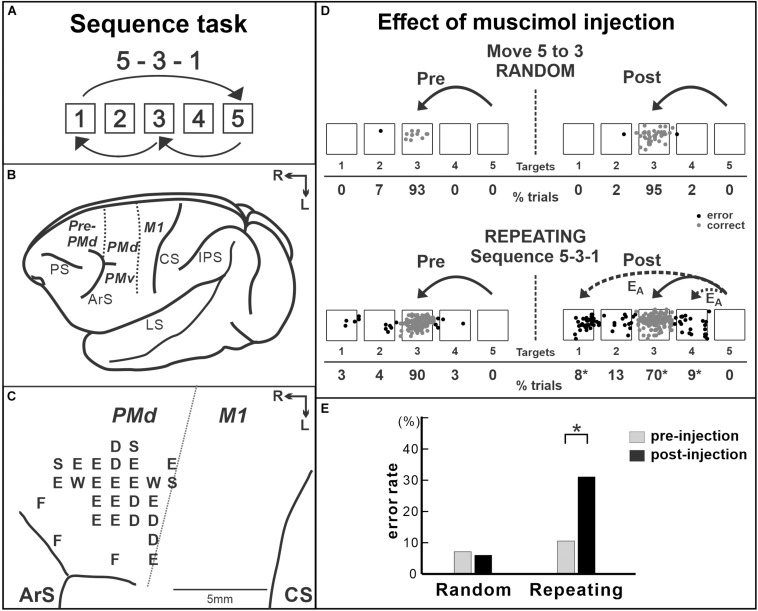
Effect of muscimol injection in the PMd on the performance of internally generated sequential movements. **(A)** Sequence 5-3-1 of the repeating task. **(B)** Lateral view of a *Cebus* brain. *Dashed lines* indicate the M1–PMd border and the pre-PMd–PMd border. *PS*, principal sulcus; *ArS*, arcuate sulcus; *CS*, central sulcus; *IPS*, intra parietal sulcus; *LS*, lateral sulcus; *pre-PMd*, pre-dorsal premotor cortex; *PMd*, dorsal premotor cortex; *M1*, primary motor cortex; *R*, rostral; *L*, lateral. **(C)** Intracortical stimulation map of a *Cebus* monkey. *Letters* indicate the movements evoked at each site. *S*, shoulder; *E*, elbow; *W*, wrist; *D*, digit; *F*, face. Injections were done at sites in which intracortical stimulation evoked shoulder or elbow movements (i.e., arm representation) in the PMd. **(D)** Reaching end points of movements from target 5 to target 3 before and after muscimol injection in the PMd. *Left*: pre-injection; *right*: post-injection. *Top*: random task; *bottom*: repeating task. The monkey was performing sequence 5-3-1 during the repeating task. *E*_*A*_: accuracy errors, a reach performed in the correct direction (e.g., to the left), but to an end point outside of the correct target. *Gray dots*: correct response; *black dots*: error response. The percentages of trials ending in each target are given *below the targets*. *p < 0.05. **(E)** Error rates of the random task (*left*) and the repeating task (*Right*) in the injection session in **(D)**. After muscimol injection, the number of errors increased dramatically in the repeating task, but not in the random task (modified from Ohbayashi et al., 2016. Copyright 2016 Society for Neuroscience).

Moreover, human imaging studies consistently reported the activity of the PMd during the performance of sequential movements (Dayan and Cohen, 2011; Hardwick et al., 2013). The studies indicated that the PMd may be a structure of key importance for sequence learning and may contribute to sequence learning by selecting appropriate responses. This idea was verified by a study using non-human primates. The role of the PMd in internally guided sequential reaching was studied using neural recordings and local inactivation (Ohbayashi et al., 2016). Monkeys were trained to perform two types of reaching tasks (Figures 2A–C). In one task, the movements were instructed by spatial visual cues (random task, visually guided reaching; Figure 2B), whereas in the other task, sequential movements were internally generated from memory after extended practice (repeating task, internally generated sequential movements guided by memory; Figure 2C; Ohbayashi et al., 2016; Ohbayashi and Picard, 2020). After more than 50 days of training on the tasks, the group examined neural activity in the arm area of the PMd, which was identified by intracortical microstimulation. About 40% of the neurons displayed responses that were enhanced in one task compared with the other (i.e., differential neurons). Approximately half of the differential neurons displayed enhanced activity during the repeating task, internally generated sequential movements. In the same study, the PMd was locally and transiently inactivated by injecting a small amount of muscimol into the arm representation area of the PMd after more than 50 days of training (Figures 3A–E). The inactivation of the PMd had a marked effect only on the performance of sequential movements that were guided by memory, but not on the performance of visually guided reaching (Figures 3D,E; Ohbayashi et al., 2016). Even though comparable numbers of neurons displayed enhanced activity during the internally guided sequential reaching and visually guided reaching, movement performance during the visually guided reaching was unaffected by the PMd inactivation. Furthermore, the monkeys made two types of errors after the inactivation of the PMd: errors of accuracy and errors in direction. Accuracy errors reveal an execution deficit: the monkeys reached in the correct direction for the next target in the sequence, but the movement end points were outside of the correct target. Direction errors indicate a deficit in the selection of the next target in a sequence: the monkeys reached in the direction opposite to the correct target. The inactivation results provide a clear demonstration of the importance of the PMd in the performance of internally generated sequential movements. Similarly, an inactivation of the left PMd of a human subject using transcranial magnetic stimulation (TMS) disrupted the performance of internally generated sequential movements (Wymbs and Grafton, 2013). In the study, human subjects practiced sequence production task using either a button box or a laptop keyboard with their right hand. After 30 days of practice, when the left PMd was stimulated, the error rate during the retrieval of practiced sequences increased.

Taken together, the results suggest that, although the PMd neurons are active during both visually guided and internally generated sequential movements, the PMd plays an important role in the internal generation of sequential movements. The inactivation results demonstrated that the PMd is involved in guiding sequential movements based on internal instructions after practice. With practice on sequential movements, the animal could learn arbitrary motor–motor associations of elements in the sequence and perform the practiced sequence in a seamless and predictive manner. Therefore, one possible interpretation is that the PMd inactivation disrupted the arbitrary motor–motor associations in the same way as lesions of the premotor cortex disrupt an animal’s performance of arbitrary sensorimotor associations (Halsband and Passingham, 1982; Wise, 1985; Passingham, 1988; di Pellegrino and Wise, 1993; Kurata and Hoffman, 1994). This is consistent with human imaging studies in which performance of the serial reaction time task (SRTT) variants elicited the bilateral PMd activity (e.g., Hardwick et al., 2013). Hardwick et al. (2013) suggested that the left PMd of humans is “a critical node in the motor learning network” for sequential movements. Further studies are necessary to explore the role of the PMd during early learning and after extensive practice, as well as in different types of sequential movements such as non-spatial sequence tasks.

## Primary Motor Cortex

The primary motor cortex (M1) controls muscle activity through its projections to the spinal cord, and its contribution to patterning muscle activity has been extensively studied (Evarts, 1981). Growing evidence suggests that M1 is involved in both the learning and maintenance of motor skills (e.g., Shibasaki et al., 1993; Karni et al., 1995, 1998; Ungerleider et al., 2002; Floyer-Lea and Matthews, 2004). For example, human imaging studies have shown that the fMRI blood oxygen level-dependent (BOLD) signal in M1 is modulated by the learning of sequential movement tasks. Karni et al. (1995) reported that after 3 weeks of practice on finger opposition sequences, the extent of M1 activation evoked during the performance of a trained sequence was significantly larger compared with the extent of activation evoked by the control task. The change in the BOLD signal in M1 persisted for several months. Moreover, the effects of prolonged and repetitive practice on the functional organization and cortical structure in M1 have been studied with musicians (i.e., the experts of sequential movements). The functional activation in M1 during the performance of sequential tasks is reduced or becomes more focused in professional musicians compared to non-musicians or amateurs (Hund-Georgiadis and von Cramon, 1999; Jancke et al., 2000; Krings et al., 2000; Haslinger et al., 2004; Meister et al., 2005). The reduced activation after years of extensive training is considered as evidence for the increased efficacy of the motor system and the need for a smaller number of active neurons to perform a highly trained set of sequential movements (Jancke et al., 2000; Krings et al., 2000; Haslinger et al., 2004; Meister et al., 2005). These suggested that the M1 of musicians is reorganized after years of extensive practice on sequential movements.

The view that M1 is reorganized after extensive practice on sequential movements has also been supported by studies focused on the anatomical and functional changes of musicians’ M1. The volume of M1 is reported to be larger in professional musicians compared to that in amateurs or non-musicians (Amunts et al., 1997; Gaser and Schlaug, 2003a,b; Draganski and May, 2008; Herholz and Zatorre, 2012; Zatorre et al., 2012; Sampaio-Baptista and Johansen-Berg, 2017; Wenger et al., 2017). The motor representations of the body parts used for skilled performance are enlarged in professional musicians compared with non-musicians (Elbert et al., 1995; Schwenkreis et al., 2007). The structural changes were proposed to be supported by processes occurring at the synapse level, including intracortical remodeling of dendritic spines and axonal terminals, glial hypertrophy, and synaptogenesis (Anderson et al., 1994; Draganski and May, 2008; Herholz and Zatorre, 2012; Zatorre et al., 2012). These studies suggested that increased synaptic efficacy as a result of extensive practice may contribute to changes in structural volume. Similarly, the plasticity of the white matter structure was correlated with skill practice, such as the number of practice hours (Bengtsson et al., 2005; Han et al., 2009). Bengtsson et al. (2005) discussed that increased myelination, caused by neural activity in fiber tracts during training, could be a mechanism underlying the observed increased volume of white matter. Taken together, extensive practice on sequential movements is suggested to lead to the increased synaptic efficacy in M1 through the remodeling of dendritic spines and axonal terminals, synaptogenesis, increased myelination, and glial hypertrophy. The change of fMRI activation in the M1 of humans, decreased 2DG signal in the M1 of non-human primates, and the enlarged volume of the M1 in musicians may all reflect the reorganization in M1 with extensive practice.

Recent fMRI studies suggested that M1’s contribution to structured and higher-order aspects of sequential movements may be limited when the training duration was short (Yokoi and Diedrichsen, 2019). In the study, human subjects practiced higher-order sequences that are composed of chunks of short sequences of keyboard pressing. Then, the authors examined whether this hierarchical structure was reflected in the brain activity patterns of the participants using fMRI data (Yokoi and Diedrichsen, 2019). The authors concluded that single-finger movements were represented in M1 and higher-order sequences were represented throughout the frontoparietal regions of the cortex after 1 week of training. The neural basis for the acquisition and retention of long and high-ordered sequences after extensive practice should be further investigated in future studies.

Neurophysiological studies in non-human primates showed that the neural activity in M1 is modulated by the sequence components. When a monkey performs sequential movements, the neural activity of M1 neurons reflects aspects of the sequential movements (Hatsopoulos et al., 2003; Lu and Ashe, 2005). The effect of extensive practice on the neural and metabolic activities in the M1 of monkeys was examined after 1–6 years of training on a sequential reaching task (Matsuzaka et al., 2007; Picard et al., 2013). In these studies, the monkeys were trained to perform the internally generated sequential reaching task and visually guided reaching task for 1–6 years (Figure 2). Then, the neural and metabolic activities were compared between these two conditions to elucidate the characteristics specific to the extensively trained sequential movements. After extensive training on the two tasks (∼2 years), Matsuzaka et al. (2007) recorded the activity of single neurons in the proximal arm representation of M1. In this experiment design, the movements were performed either in the context of an internally generated trained sequence or of a visually guided reaching on the same experiment day (e.g., movement from target 5 to target 3 in Figures 2B,C). Therefore, the comparison of activity for movements performed in two different contexts (i.e., internally generated sequence or visually guided reaching) revealed changes of activity associated with training, even though the activity patterns of the neurons before training were unknown (i.e., not recorded). Forty percent of the task-related neurons in M1 were differentially active during the performance of the visually guided and internally generated sequential reaching. The majority of differentially active neurons had enhanced activity for the trained, internally generated sequential reaching (Matsuzaka et al., 2007). Similarly, the uptake of 2DG was examined in the arm area of M1 after extensive training of the sequential reaching task (Picard et al., 2013). Uptake of 2DG is suggested to be associated with presynaptic activity at both excitatory and inhibitory synapses (discussed in Picard and Strick, 2003; Picard et al., 2013). They found that the uptake of 2DG was low in monkeys that performed highly practiced, internally generated sequences of movements compared with the 2DG uptake in monkeys that performed visually guided reaching (Picard et al., 2013). Surprisingly, the low uptake of 2DG was not matched by low neural activity in the same area. Neural activity in arm M1 during the internally generated movements was comparable to that observed during the visually guided movements. Therefore, there was a marked dissociation between the metabolic and neural activities in M1. These observations imply an increase of the synaptic efficacy in M1 after extensive practice, which led to M1’s contribution to the planning and generation of sequential movements.

**FIGURE 4:**
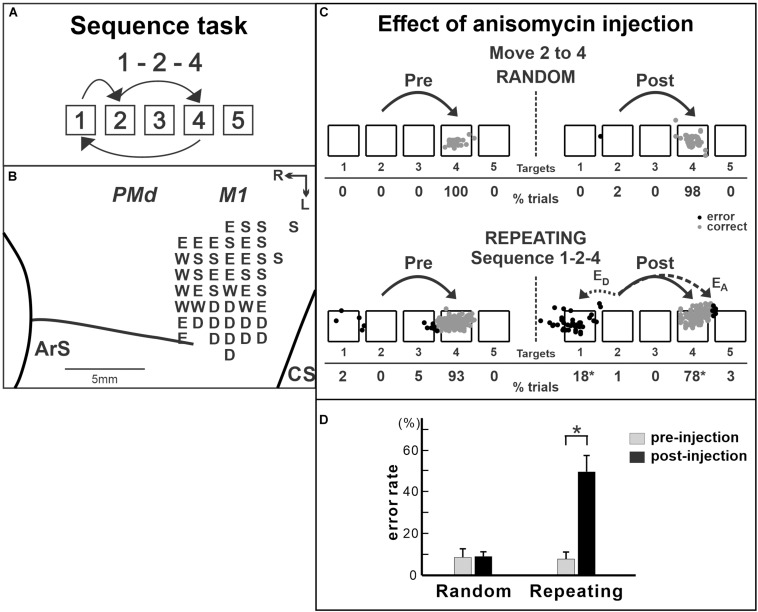
Effect of anisomycin injection in M1 on the performance of internally generated sequential movements. **(A)** Sequence 1-2-4 of the repeating task. **(B)** Intracortical stimulation map of a *Cebus* monkey. *Letters* indicate the movements evoked at each site. *S*, shoulder; *E*, elbow; *W*, wrist; *D*, digit. Injections were done at sites in which intracortical stimulation evoked shoulder or elbow movements (i.e., arm representation). **(C)** Reaching end points of trials from target 2 to target 4 before and after anisomycin injection. *Left*: pre-injection; *right*: post-injection. *Top*: random task; *bottom*: repeating task. The monkey was performing sequence 1-2-4 during the repeating task. *E*_*A*_: accuracy errors, a reach performed in the correct direction (e.g., to the right), but to an end point outside of the correct target; *E*_*D*_: direction errors, a reach performed in the direction opposite to the correct target. *Gray dots*: correct response; *black dots*: error response. The percentages of trials ending in each target are given *below the targets*. *p < 0.05. **(D)** Averaged error rates of six injection sessions in the random task (*left*) and the repeating task (*right*). After anisomycin injection, the number of errors increased dramatically in the repeating task, but not in the random task (modified from Ohbayashi, 2020).

M1 is critical for implementing motor output, so that it has been challenging to test its involvement in the acquisition or the maintenance of motor sequences. Lesions or inactivation of M1 will abolish the motor commands to the spinal cord that generates muscle activity. A few studies reported that, when M1 was inactivated, subjects made more errors in the performance of trained sequential movements compared with that before the inactivation (Lu and Ashe, 2005; Cohen et al., 2009; Censor et al., 2014). However, because M1 is critically involved in motor execution, an advanced approach is required to further understand how M1 contributes to internally generated sequential movements without the confound of basic motor deficits. This was achieved in a recent study by selectively manipulating protein synthesis in the M1 of non-human primates in order to disrupt information storage in this cortical area (Figure 4; Ohbayashi, 2020). In the study, the monkeys were trained on two tasks: internally generated sequential movements (repeating task, guided by memory; Figures 2C, 4A) and reaching movements guided by visual cues (random task, visually guided reaching as a control task; Figure 2B; Ohbayashi, 2020). After the monkeys practiced each sequence for more than 100 training days and started to perform the memorized sequential movements predictively, the protein synthesis inhibitor anisomycin was injected into the arm representation of M1 to test M1’s involvement in the maintenance of sequential movements after extensive practice (Figure 4B). Anisomycin injections had a significant effect on the performance of the sequential movements guided by memory during the repeating task. The injections resulted in a significant increase in the number of errors (Figures 4C,D) and a significant decrease in the number of predictive responses, an indication of sequence learning, during the repeating task. Moreover, the monkeys made errors reaching in the direction opposite to the correct target (Figure 4C, bottom). This type of error suggests a deficit in selecting the movement component in the sequence. In contrast, performance of the visually guided movements during the random task was not significantly disrupted. Interestingly, inactivation of M1 using muscimol injection disrupted the performance on both the random and repeating tasks, suggesting that the inactivation of M1 caused a deficit of motor production (Ohbayashi, 2020). Differences in the effects between anisomycin injection and muscimol injection suggest that the anisomycin injection disrupted the performance of internally generated sequential movements by interfering with the information storage in this area. This observation emphasizes the importance of M1 for the generation of sequential movements guided by memory. The results suggest that, although M1 is critical for movement production, it also is involved in the maintenance of skilled sequential movements (Ohbayashi, 2020).

Protein synthesis inhibitors have been widely used in rodents to study the neural basis of learning and memory. The studies have been conducted in rodents, especially extensively in the context of fear conditioning (Davis and Squire, 1984; Nader et al., 2000a,b; Kandel, 2001; Dudai, 2004, 2012; Dudai and Eisenberg, 2004; Kelleher et al., 2004; Rudy, 2008a,b). *De novo* protein synthesis, during or shortly after the initial training, is shown to be essential in the consolidation of long-term memory (Davis and Squire, 1984). Moreover, when a protein synthesis inhibitor (e.g., anisomycin) was given during the retrieval, the performance of retrieved task was disrupted (Nader et al., 2000a; Nader, 2003; Lee et al., 2008). The studies suggested that the neural trace may be destabilized upon retrieval through protein degradation and then rebounded through protein synthesis during the “reconsolidation” process (Nader et al., 2000a,b; Sara, 2000; Nader, 2003; Lee et al., 2008; Rudy, 2008a,b; Dudai, 2012). Thus, the injection of the protein synthesis inhibitor disrupted the task performance as the inhibitor prevented the synthesis of the proteins needed to reconsolidate the memory trace (Nader et al., 2000a; Lee et al., 2008; Dudai, 2012). The series of studies also proposed that the destabilized trace may be bidirectionally modified to be weakened or strengthened, so that the neural trace can be “updated” (Sara, 2000; Dudai and Eisenberg, 2004; Rudy, 2008b; Dudai, 2012). Although it is unclear whether these proposals can be generalized to other forms of memory, they may inform us of the way by which anisomycin injected in M1 interfered with the performance of the well-practiced sequential movements (discussed in Ohbayashi, 2020). The neural basis for motor skill improvement needs to be further investigated in future studies.

The rodent studies provide valuable insights into the reorganization of the motor cortex during motor skill learning, even though the rodent motor system and the range of motor skills differ from those of human and non-human primates (Dum and Strick, 1991; He et al., 1993, 1995; Rathelot et al., 2016). Early in the learning of the reach–grasp task, the expressions of transcription factors (e.g., an immediate early gene, *c-fos*) increase within the rodent’s motor cortex and remain elevated in the plateau phase of the learning curve relative to control animals (Kleim et al., 1996). The increase of gene expression precedes both the changes in synapse number and motor map reorganization (Kleim et al., 1996). The injection of protein synthesis inhibitors into the motor cortex of rodents disrupted the maintenance (Kleim et al., 2003) or the learning (Luft et al., 2004) of the skilled forelimb reaching. In these studies, the rats were trained to reach and grasp for a food pellet placed outside the cage (Kleim et al., 2003; Luft et al., 2004). The injection of anisomycin into the motor cortex after the training disrupted the performance of the skilled forelimb task and caused reductions in the synapse number and size in the motor cortex *in vivo* (Kleim et al., 2003). The injection of anisomycin into the rodents’ motor cortex during the learning disrupted the learning of the motor skill task (Luft et al., 2004). Two photon imaging or electron microscopy studies have shown that skill training leads to the rapid formation of enduring postsynaptic dendritic spines and an increase in synaptic density in neurons in the motor cortex (Kleim et al., 2003; Xu et al., 2009; Yang et al., 2009; Yu and Zuo, 2011). When the newly modified dendritic spines during the training of rotarod tasks were optically manipulated to shrink in the motor cortex, the rodents’ performance of the trained task was disrupted (Hayashi-Takagi et al., 2015). The study suggested that the structural plasticity of spines plays a critical role in the learning of motor skills in the motor cortex of rodents.

Taken together, these observations support the view that M1 is involved in skilled sequential movements, especially after extensive practice. The neural activity, metabolic activity, and the structural organization in M1 were influenced by extensive practice on the motor skill tasks. Further studies will expand our understanding of how M1 contributes to the continuous improvement of skilled sequential movements during repetitive practice as well as its contribution to fast learning.

## Collaboration of Cortical Motor Areas

Studies on anatomical connectivity provided valuable insights into the interaction between multiple areas. The functional distinction of pre-SMA and SMA is supported by the differences in the anatomical connections between these areas (Luppino et al., 1990, 1993; Bates and Goldman-Rakic, 1993, reviewed in Picard and Strick, 2001). Firstly, only the SMA has direct projections to the M1 and the spinal cord (Muakkassa and Strick, 1979; Dum and Strick, 1991, 1996; He et al., 1995; Wang et al., 2001). Secondly, the pre-SMA does not have substantial connections with M1 (Tokuno and Tanji, 1993; Galea and Darian-Smith, 1994; Lu et al., 1994; Hatanaka et al., 2001; Dum and Strick, 2005). Instead, the pre-SMA is densely interconnected with regions of the prefrontal cortex as well as from the rostral cingulate motor area and pre-PMd (F7) (Luppino et al., 1990, 1993, 2003; Bates and Goldman-Rakic, 1993; Lu et al., 1994; Takada et al., 2004; Wang et al., 2005). Moreover, the pre-SMA does not appear to be densely interconnected with the SMA (Luppino et al., 1990, 1993, 2003; Wang et al., 2001). These observations suggest that the SMA is a part of the cortical motor areas and that the pre-SMA can be functionally considered as a region of the prefrontal areas (Bates and Goldman-Rakic, 1993; Luppino et al., 1993; Lu et al., 1994; Picard and Strick, 2001). This view is consistent with the observations of the inactivation studies described above showing that the pre-SMA is involved in cognitive aspects such as the early learning of movement sequences, whereas the SMA is primarily involved in the performance of memorized movement sequences.

The anatomical connections of the M1, PMd, PMv (ventral premotor cortex), and the SMA of monkeys were precisely studied by Dum and Strick’s group (Dum and Strick, 2005). The anatomy results showed that the digit representations of the PMd, PMv, and M1 are densely interconnected with each other. Thus, these three cortical areas form a network for the control of hand movements (Dum and Strick, 2005). The projections from the digit representation in the SMA to the PMd and the PMv are stronger than the SMA projections to M1 (Dum and Strick, 2005). This suggests that the SMA may influence through connections with the premotor areas rather than through M1. Overall, the laminar origins of neurons that interconnect the PMd, PMv, and M1 are typical of “lateral” interactions. Dum and Strick commented that “from an anatomical perspective, this cortical network lacks a clear hierarchical organization” (Dum and Strick, 2005). The strong, reciprocal interconnections suggest that these areas may act in concert with each other to produce commands for movements.

In fact, a subset of neurons in each premotor area exhibits activity for relatively simple movements as M1 neurons do (Kurata and Tanji, 1986; Shima et al., 1991; Cadoret and Smith, 1997). Furthermore, in non-human primate studies, aspects of practiced sequences were reflected in the neural or the metabolic activity in all the SMA, PMd, and M1 (Picard and Strick, 1997, 2003; Matsuzaka et al., 2007; Picard et al., 2013; Ohbayashi et al., 2016; Ohbayashi, 2020). On the other hand, the injection of chemical agents in these areas showed that each premotor area is differentially involved in sequential movements. Inactivation of the SMA did not have an effect on the learning and performance of internally generated spatial sequences (Nakamura et al., 1999). Nevertheless, both the muscimol injection in the PMd and the anisomycin injection in M1 selectively disrupted the performance of internally generated sequences, but not the visually guided reaching (Ohbayashi et al., 2016; Ohbayashi, 2020). Moreover, both injections caused deficits in target selection, in which a monkey reached to the direction opposite to the correct target (Ohbayashi et al., 2016; Ohbayashi, 2020). Together with the dense anatomical connection between the PMd and M1, as described above, these suggested a possibility that anisomycin injection disrupted the interaction from the PMd to M1, which resulted in the deficit in the performance of internally generated spatial sequences. These suggest that the PMd functions as a major source of input to M1 to guide the performance of internally generated spatial sequences after practice. More experiments are required to tease out the exact nature of interactions between M1 and the premotor areas in the learning and performance of sequential movements.

## Summary

The performance of sequential movements can be improved to the expert level and maintained as a motor skill through extensive practice. Functional imaging studies in humans show that a brain-wide network subserves the performance of skilled sequential movements. Interventional studies in non-human primates advanced our understanding of its neural basis. The results of interventional studies suggested that each motor area in the network makes a distinct contribution to skilled sequential movements. The SMA is involved in the temporal organization of multiple non-spatial movements into a sequence and the execution of the sequential actions. Its role in spatial sequences is still debatable and needs to be further investigated. The PMd may act as a key structure for the learning of sequential movements by contributing to the selection of appropriate responses. Specifically, the PMd may be critical for the acquisition and maintenance of arbitrary motor–motor associations. In M1, the neural activity, metabolic activity, and structural organization were shown to be modified by extensive practice on sequential movements. M1’s involvement in sequential movements after extensive practice was verified by an interventional study using an inhibitor for protein synthesis. These studies suggest that the PMd functions as a major source of input to M1 to guide the performance of internally generated sequences. Together, the PMd and M1 may be parts of the key structures for the learning and maintenance of internally generated sequential movements. The involvements of these areas along the dimensions of time (i.e., learning stages) and sequence category (e.g., spatial and non-spatial) need to be further explored in future experiments.

## Author Contributions

The author confirms being the sole contributor of this work and has approved it for publication.

## Conflict of Interest

The author declares that the research was conducted in the absence of any commercial or financial relationships that could be construed as a potential conflict of interest.

## References

[B1] Amunts K. Schlaug G. Jancke L. Steinmetz H. Schleicher A. Dabringhaus A. (1997). Motor cortex and hand motor skills: structural compliance in the human brain. *Hum. Brain Mapp.* 5 206–215. 10.1002/(SICI)1097-019319975:3<206::AID-HBM5>3.0.CO;2-7 20408216

[B2] Anderson B. J. Li X. Alcantara A. A. Isaacs K. R. Black J. E. Greenough W. T. (1994). Glial hypertrophy is associated with synaptogenesis following motor-skill learning, but not with angiogenesis following exercise. *Glia* 11 73–80. 10.1002/glia.440110110 7520887

[B3] Averbeck B. B. Battaglia-Mayer A. Guglielmo C. Caminiti R. (2009). Statistical analysis of parieto-frontal cognitive-motor networks. *J. Neurophysiol.* 102 1911–1920. 10.1152/jn.00519.2009 19625537

[B4] Bassett D. S. Yang M. Wymbs N. F. Grafton S. T. (2015). Learning-induced autonomy of sensorimotor systems. *Nat. Neurosci.* 18 744–751. 10.1038/nn.3993 25849989 PMC6368853

[B5] Bates J. F. Goldman-Rakic P. S. (1993). Prefrontal connections of medial motor areas in the rhesus monkey. *J. Comp. Neurol.* 336 211–228. 10.1002/cne.903360205 7503997

[B6] Bengtsson S. L. Nagy Z. Skare S. Forsman L. Forssberg H. Ullen F. (2005). Extensive piano practicing has regionally specific effects on white matter development. *Nat. Neurosci.* 8 1148–1150. 10.1038/nn1516 16116456

[B7] Boecker H. Dagher A. Ceballos-Baumann A. O. Passingham R. E. Samuel M. Friston K. J. (1998). Role of the human rostral supplementary motor area and the basal ganglia in motor sequence control: investigations with H2 15O PET. *J. Neurophysiol.* 79 1070–1080. 10.1152/jn.1998.79.2.1070 9463462

[B8] Brinkman C. (1984). Supplementary motor area of the monkey’s cerebral cortex: short- and long-term deficits after unilateral ablation and the effects of subsequent callosal section. *J. Neurosci.* 4 918–929. 10.1523/JNEUROSCI.04-04-00918.1984 6716131 PMC6564786

[B9] Cadoret G. Smith A. M. (1997). Comparison of the neuronal activity in the SMA and the ventral cingulate cortex during prehension in the monkey. *J. Neurophysiol.* 77 153–166. 10.1152/jn.1997.77.1.153 9120556

[B10] Censor N. Horovitz S. G. Cohen L. G. (2014). Interference with existing memories alters offline intrinsic functional brain connectivity. *Neuron* 81 69–76. 10.1016/j.neuron.2013.10.042 24411732 PMC3894578

[B11] Cisek P. Kalaska J. F. (2002). Simultaneous encoding of multiple potential reach directions in dorsal premotor cortex. *J. Neurophysiol.* 87 1149–1154. 10.1152/jn.00443.2001 11826082

[B12] Cisek P. Kalaska J. F. (2004). Neural correlates of mental rehearsal in dorsal premotor cortex. *Nature* 431 993–996. 10.1038/nature03005 15496925

[B13] Cisek P. Kalaska J. F. (2005). Neural correlates of reaching decisions in dorsal premotor cortex: specification of multiple direction choices and final selection of action. *Neuron* 45 801–814. 10.1016/j.neuron.2005.01.027 15748854

[B14] Clower W. T. Alexander G. E. (1998). Movement sequence-related activity reflecting numerical order of components in supplementary and presupplementary motor areas. *J. Neurophysiol.* 80 1562–1566. 10.1152/jn.1998.80.3.1562 9744961

[B15] Cohen N. R. Cross E. S. Wymbs N. F. Grafton S. T. (2009). Transient disruption of M1 during response planning impairs subsequent offline consolidation. *Exp. Brain Res.* 196 303–309. 10.1007/s00221-009-1838-x 19462166 PMC2693775

[B16] Cona G. Semenza C. (2017). Supplementary motor area as key structure for domain-general sequence processing: a unified account. *Neurosci. Biobehav. Rev.* 72 28–42. 10.1016/j.neubiorev.2016.10.033 27856331

[B17] Coynel D. Marrelec G. Perlbarg V. Pelegrini-Issac M. van de Moortele P. F. Ugurbil K. (2010). Dynamics of motor-related functional integration during motor sequence learning. *Neuroimage* 49 759–766. 10.1016/j.neuroimage.2009.08.048 19716894 PMC2764831

[B18] Davis H. P. Squire L. R. (1984). Protein synthesis and memory: a review. *Psychol. Bull.* 96 518–559. 10.1037/0033-2909.96.3.518 6096908

[B19] Dayan E. Cohen L. G. (2011). Neuroplasticity subserving motor skill learning. *Neuron* 72 443–454. 10.1016/j.neuron.2011.10.008 22078504 PMC3217208

[B20] di Pellegrino G. Wise S. P. (1993). Effects of attention on visuomotor activity in the premotor and prefrontal cortex of a primate. *Somatosens. Mot. Res.* 10 245–262. 10.3109/08990229309028835 8237213

[B21] Dick J. P. Benecke R. Rothwell J. C. Day B. L. Marsden C. D. (1986). Simple and complex movements in a patient with infarction of the right supplementary motor area. *Mov. Disord.* 1 255–266. 10.1002/mds.870010405 3504248

[B22] Doyon J. Benali H. (2005). Reorganization and plasticity in the adult brain during learning of motor skills. *Curr. Opin. Neurobiol.* 15 161–167. 10.1016/j.conb.2005.03.004 15831397

[B23] Doyon J. Penhune V. Ungerleider L. G. (2003). Distinct contribution of the cortico-striatal and cortico-cerebellar systems to motor skill learning. *Neuropsychologia* 41 252–262. 10.1016/s0028-3932(02)00158-6 12457751

[B24] Draganski B. May A. (2008). Training-induced structural changes in the adult human brain. *Behav. Brain Res.* 192 137–142. 10.1016/j.bbr.2008.02.015 18378330

[B25] Dudai Y. (2004). The neurobiology of consolidations, or, how stable is the engram? *Annu. Rev. Psychol.* 955 51–86. 10.1146/annurev.psych.55.090902.142050 14744210

[B26] Dudai Y. (2012). The restless engram: consolidations never end. *Annu. Rev. Neurosci.* 35 227–247. 10.1146/annurev-neuro-062111-150500 22443508

[B27] Dudai Y. Eisenberg M. (2004). Rites of passage of the engram: reconsolidation and the lingering consolidation hypothesis. *Neuron* 44 93–100. 10.1016/j.neuron.2004.09.003 15450162

[B28] Dum R. P. Strick P. L. (1991). The origin of corticospinal projections from the premotor areas in the frontal lobe. *J. Neurosci.* 11 667–689. 10.1523/JNEUROSCI.11-03-00667.1991 1705965 PMC6575356

[B29] Dum R. P. Strick P. L. (1996). Spinal cord terminations of the medial wall motor areas in macaque monkeys. *J. Neurosci.* 16 6513–6525. 10.1523/JNEUROSCI.16-20-06513.1996 8815929 PMC6578918

[B30] Dum R. P. Strick P. L. (2005). Frontal lobe inputs to the digit representations of the motor areas on the lateral surface of the hemisphere. *J. Neurosci.* 25 1375–1386. 10.1523/JNEUROSCI.3902-04.2005 15703391 PMC6726000

[B31] Elbert T. Pantev C. Wienbruch C. Rockstroh B. Taub E. (1995). Increased cortical representation of the fingers of the left hand in string players. *Science* 270 305–307. 10.1126/science.270.5234.305 7569982

[B32] Evarts E. V. (1981). “Chapter 23: role of motor cortex in voluntary movements in primates,” in *Handbook of Physiology, The Nervous System, Motor Control*, Vol. 2 ed. Brook V. B. (Baltimore, MD: Williams and Wilkins).

[B33] Fitts P. M. Posner M. I. (1967). *Human Performance.* Belmont, CA: Brooks/Cole Publishers.

[B34] Floyer-Lea A. Matthews P. M. (2004). Changing brain networks for visuomotor control with increased movement automaticity. *J. Neurophysiol.* 92 2405–2412. 10.1152/jn.01092.2003 15381748

[B35] Galea M. P. Darian-Smith I. (1994). Multiple corticospinal neuron populations in the macaque monkey are specified by their unique cortical origins, spinal terminations, and connections. *Cereb. Cortex* 4 166–194. 10.1093/cercor/4.2.166 8038567

[B36] Gaser C. Schlaug G. (2003a). Brain structures differ between musicians and non-musicians. *J. Neurosci.* 23 9240–9245. 10.1523/JNEUROSCI.23-27-09240.2003 14534258 PMC6740845

[B37] Gaser C. Schlaug G. (2003b). Gray matter differences between musicians and non-musicians. *Ann. N. Y. Acad. Sci.* 999 514–517. 10.1196/annals.1284.062 14681175

[B38] Gerloff C. Corwell B. Chen R. Hallett M. Cohen L. G. (1997). Stimulation over the human supplementary motor area interferes with the organization of future elements in complex motor sequences. *Brain* 120(Pt 9) 1587–1602. 10.1093/brain/120.9.1587 9313642

[B39] Goldberg G. (1985). Supplementary motor area structure and function – review and hypotheses. *Behav. Brain Sci.* 8 567–588. 10.1017/S0140525x00045167

[B40] Grafton S. T. Hazeltine E. Ivry R. (1995). Functional mapping of sequence learning in normal humans. *J. Cogn. Neurosci.* 7 497–510. 10.1162/jocn.1995.7.4.497 23961907

[B41] Grafton S. T. Woods R. P. Tyszka M. (1994). Functional imaging of procedural motor learning: relating cerebral blood flow with individual subject performance. *Hum. Brain Mapp.* 1 221–234. 10.1002/hbm.460010307 24578042

[B42] Halsband U. (1987). “Higher disturbances of movement in monkeys (*Macaca mulatta*),” in *Motor Control*, eds Gantchev G. N. Dimitrov B. Gatev P. (New York, NY: Plenum Press), 79–85. 10.1007/978-1-4615-7508-5_14

[B43] Halsband U. Ito N. Tanji J. Freund H. J. (1993). The role of premotor cortex and the supplementary motor area in the temporal control of movement in man. *Brain* 116(Pt 1) 243–266. 10.1093/brain/116.1.243 8453461

[B44] Halsband U. Passingham R. (1982). The role of premotor and parietal cortex in the direction of action. *Brain Res.* 240 368–372. 10.1016/0006-8993(82)90239-6 7104700

[B45] Han Y. Yang H. Lv Y. T. Zhu C. Z. He Y. Tang H. H. (2009). Gray matter density and white matter integrity in pianists’ brain: a combined structural and diffusion tensor MRI study. *Neurosci. Lett.* 459 3–6. 10.1016/j.neulet.2008.07.056 18672026

[B46] Hardwick R. M. Rottschy C. Miall R. C. Eickhoff S. B. (2013). A quantitative meta-analysis and review of motor learning in the human brain. *Neuroimage* 67 283–297. 10.1016/j.neuroimage.2012.11.020 23194819 PMC3555187

[B47] Haslinger B. Erhard P. Altenmuller E. Hennenlotter A. Schwaiger M. Grafin Von Einsiedel H. (2004). Reduced recruitment of motor association areas during bimanual coordination in concert pianists. *Hum. Brain Mapp.* 22 206–215. 10.1002/hbm.20028 15195287 PMC6871883

[B48] Hatanaka N. Nambu A. Yamashita A. Takada M. Tokuno H. (2001). Somatotopic arrangement and corticocortical inputs of the hindlimb region of the primary motor cortex in the macaque monkey. *Neurosci. Res.* 40 9–22. 10.1016/s0168-0102(01)00210-3 11311401

[B49] Hatsopoulos N. G. Paninski L. Donoghue J. P. (2003). Sequential movement representations based on correlated neuronal activity. *Exp. Brain Res.* 149 478–486. 10.1007/s00221-003-1385-9 12677328

[B50] Hayashi-Takagi A. Yagishita S. Nakamura M. Shirai F. Wu Y. I. Loshbaugh A. L. (2015). Labelling and optical erasure of synaptic memory traces in the motor cortex. *Nature* 525 333–338. 10.1038/nature15257 26352471 PMC4634641

[B51] He S. Q. Dum R. P. Strick P. L. (1993). Topographic organization of corticospinal projections from the frontal lobe: motor areas on the lateral surface of the hemisphere. *J. Neurosci.* 13 952–980. 10.1523/JNEUROSCI.13-03-00952.1993 7680069 PMC6576595

[B52] He S. Q. Dum R. P. Strick P. L. (1995). Topographic organization of corticospinal projections from the frontal lobe: motor areas on the medial surface of the hemisphere. *J. Neurosci.* 15 3284–3306. 10.1523/JNEUROSCI.15-05-03284.1995 7538558 PMC6578253

[B53] Herholz S. C. Zatorre R. J. (2012). Musical training as a framework for brain plasticity: behavior, function, and structure. *Neuron* 76 486–502. 10.1016/j.neuron.2012.10.011 23141061

[B54] Hikosaka O. Nakamura K. Sakai K. Nakahara H. (2002). Central mechanisms of motor skill learning. *Curr. Opin. Neurobiol.* 12 217–222. 10.1016/s0959-4388(02)00307-0 12015240

[B55] Hikosaka O. Sakai K. Miyauchi S. Takino R. Sasaki Y. Putz B. (1996). Activation of human presupplementary motor area in learning of sequential procedures: a functional MRI study. *J. Neurophysiol.* 76 617–621. 10.1152/jn.1996.76.1.617 8836248

[B56] Hoshi E. Tanji J. (2007). Distinctions between dorsal and ventral premotor areas: anatomical connectivity and functional properties. *Curr. Opin. Neurobiol.* 17 234–242. 10.1016/j.conb.2007.02.003 17317152

[B57] Hund-Georgiadis M. von Cramon D. Y. (1999). Motor-learning-related changes in piano players and non-musicians revealed by functional magnetic-resonance signals. *Exp. Brain Res.* 125 417–425. 10.1007/s002210050698 10323287

[B58] Jancke L. Shah N. J. Peters M. (2000). Cortical activations in primary and secondary motor areas for complex bimanual movements in professional pianists. *Brain Res. Cogn. Brain Res.* 10 177–183. 10.1016/s0926-6410(00)00028-8 10978706

[B59] Johnson P. B. Ferraina S. Bianchi L. Caminiti R. (1996). Cortical networks for visual reaching: physiological and anatomical organization of frontal and parietal lobe arm regions. *Cereb. Cortex* 6 102–119. 10.1093/cercor/6.2.102 8670643

[B60] Kalaska J. F. Crammond D. J. (1995). Deciding not to GO: neuronal correlates of response selection in a GO/NOGO task in primate premotor and parietal cortex. *Cereb. Cortex* 5 410–428. 10.1093/cercor/5.5.410 8547788

[B61] Kandel E. R. (2001). The molecular biology of memory storage: a dialogue between genes and synapses. *Science* 294 1030–1038. 10.1126/science.1067020 11691980

[B62] Karni A. Meyer G. Jezzard P. Adams M. M. Turner R. Ungerleider L. G. (1995). Functional MRI evidence for adult motor cortex plasticity during motor skill learning. *Nature* 377 155–158. 10.1038/377155a0 7675082

[B63] Karni A. Meyer G. Rey-Hipolito C. Jezzard P. Adams M. M. Turner R. (1998). The acquisition of skilled motor performance: fast and slow experience-driven changes in primary motor cortex. *Proc. Natl. Acad. Sci. U.S.A.* 95 861–868. 10.1073/pnas.95.3.861 9448252 PMC33809

[B64] Kelleher R. J. III Govindarajan A. Tonegawa S. (2004). Translational regulatory mechanisms in persistent forms of synaptic plasticity. *Neuron* 44 59–73. 10.1016/j.neuron.2004.09.013 15450160

[B65] Kleim J. A. Bruneau R. Calder K. Pocock D. Vandenberg P. M. Macdonald E. (2003). Functional organization of adult motor cortex is dependent upon continued protein synthesis. *Neuron* 40 167–176. 10.1016/s0896-6273(03)00592-0 14527441

[B66] Kleim J. A. Lussnig E. Schwarz E. R. Comery T. A. Greenough W. T. (1996). Synaptogenesis and Fos expression in the motor cortex of the adult rat after motor skill learning. *J. Neurosci.* 16 4529–4535. 10.1523/JNEUROSCI.16-14-04529.1996 8699262 PMC6578852

[B67] Krings T. Topper R. Foltys H. Erberich S. Sparing R. Willmes K. (2000). Cortical activation patterns during complex motor tasks in piano players and control subjects. A functional magnetic resonance imaging study. *Neurosci. Lett.* 278 189–193. 10.1016/s0304-3940(99)00930-1 10653025

[B68] Kurata K. Hoffman D. S. (1994). Differential effects of muscimol microinjection into dorsal and ventral aspects of the premotor cortex of monkeys. *J. Neurophysiol.* 71 1151–1164. 10.1152/jn.1994.71.3.1151 8201409

[B69] Kurata K. Tanji J. (1986). Premotor cortex neurons in macaques: activity before distal and proximal forelimb movements. *J. Neurosci.* 6 403–411. 10.1523/JNEUROSCI.06-02-00403.1986 3950703 PMC6568527

[B70] Kurata K. Wise S. P. (1988). Premotor cortex of rhesus monkeys: set-related activity during two conditional motor tasks. *Exp. Brain Res.* 69 327–343. 10.1007/BF00247578 3345810

[B71] Lee D. Quessy S. (2003). Activity in the supplementary motor area related to learning and performance during a sequential visuomotor task. *J. Neurophysiol.* 89 1039–1056. 10.1152/jn.00638.2002 12574479

[B72] Lee S. H. Choi J. H. Lee N. Lee H. R. Kim J. I. Yu N. K. (2008). Synaptic protein degradation underlies destabilization of retrieved fear memory. *Science* 319 1253–1256. 10.1126/science.1150541 18258863

[B73] Lu M. T. Preston J. B. Strick P. L. (1994). Interconnections between the prefrontal cortex and the premotor areas in the frontal lobe. *J. Comp. Neurol.* 341 375–392. 10.1002/cne.903410308 7515081

[B74] Lu X. Ashe J. (2005). Anticipatory activity in primary motor cortex codes memorized movement sequences. *Neuron* 45 967–973. 10.1016/j.neuron.2005.01.036 15797556

[B75] Luft A. R. Buitrago M. M. Ringer T. Dichgans J. Schulz J. B. (2004). Motor skill learning depends on protein synthesis in motor cortex after training. *J. Neurosci.* 24 6515–6520. 10.1523/JNEUROSCI.1034-04.2004 15269262 PMC6729880

[B76] Luppino G. Matelli M. Camarda R. Rizzolatti G. (1993). Corticocortical connections of area F3 (SMA-proper) and area F6 (pre-SMA) in the macaque monkey. *J. Comp. Neurol.* 338 114–140. 10.1002/cne.903380109 7507940

[B77] Luppino G. Matelli M. Rizzolatti G. (1990). Cortico-cortical connections of two electrophysiologically identified arm representations in the mesial agranular frontal cortex. *Exp. Brain Res.* 82 214–218. 10.1007/BF00230855 2257908

[B78] Luppino G. Rozzi S. Calzavara R. Matelli M. (2003). Prefrontal and agranular cingulate projections to the dorsal premotor areas F2 and F7 in the macaque monkey. *Eur. J. Neurosci.* 17 559–578. 10.1046/j.1460-9568.2003.02476.x 12581174

[B79] Matsuzaka Y. Picard N. Strick P. L. (2007). Skill representation in the primary motor cortex after long-term practice. *J. Neurophysiol.* 97 1819–1832. 10.1152/jn.00784.2006 17182912

[B80] Meister I. Krings T. Foltys H. Boroojerdi B. Muller M. Topper R. (2005). Effects of long-term practice and task complexity in musicians and nonmusicians performing simple and complex motor tasks: implications for cortical motor organization. *Hum. Brain Mapp.* 25 345–352. 10.1002/hbm.20112 15852385 PMC6871746

[B81] Mitz A. R. Godschalk M. Wise S. P. (1991). Learning-dependent neuronal activity in the premotor cortex: activity during the acquisition of conditional motor associations. *J. Neurosci.* 11 1855–1872. 10.1523/JNEUROSCI.11-06-01855.1991 2045890 PMC6575410

[B82] Miyachi S. Hikosaka O. Lu X. (2002). Differential activation of monkey striatal neurons in the early and late stages of procedural learning. *Exp. Brain Res.* 146 122–126. 10.1007/s00221-002-1213-7 12192586

[B83] Muakkassa K. F. Strick P. L. (1979). Frontal lobe inputs to primate motor cortex: evidence for four somatotopically organized ‘premotor’ areas. *Brain Res.* 177 176–182. 10.1016/0006-8993(79)90928-4 115545

[B84] Mushiake H. Inase M. Tanji J. (1991). Neuronal activity in the primate premotor, supplementary, and precentral motor cortex during visually guided and internally determined sequential movements. *J. Neurophysiol.* 66 705–718. 10.1152/jn.1991.66.3.705 1753282

[B85] Mushiake H. Strick P. L. (1993). Preferential activity of dentate neurons during limb movements guided by vision. *J. Neurophysiol.* 70 2660–2664. 10.1152/jn.1993.70.6.2660 8120605

[B86] Mushiake H. Strick P. L. (1995). Pallidal neuron activity during sequential arm movements. *J. Neurophysiol.* 74 2754–2758. 10.1152/jn.1995.74.6.2754 8747231

[B87] Nachev P. Kennard C. Husain M. (2008). Functional role of the supplementary and pre-supplementary motor areas. *Nat. Rev. Neurosci.* 9 856–869. 10.1038/nrn2478 18843271

[B88] Nader K. (2003). Memory traces unbound. *Trends Neurosci.* 26 65–72. 10.1016/S0166-2236(02)00042-5 12536129

[B89] Nader K. Schafe G. E. Le Doux J. E. (2000a). Fear memories require protein synthesis in the amygdala for reconsolidation after retrieval. *Nature* 406 722–726. 10.1038/35021052 10963596

[B90] Nader K. Schafe G. E. Le Doux J. E. (2000b). The labile nature of consolidation theory. *Nat. Rev. Neurosci.* 1 216–219. 10.1038/35044580 11257912

[B91] Nakamura K. Sakai K. Hikosaka O. (1998). Neuronal activity in medial frontal cortex during learning of sequential procedures. *J. Neurophysiol.* 80 2671–2687. 10.1152/jn.1998.80.5.2671 9819272

[B92] Nakamura K. Sakai K. Hikosaka O. (1999). Effects of local inactivation of monkey medial frontal cortex in learning of sequential procedures. *J. Neurophysiol.* 82 1063–1068. 10.1152/jn.1999.82.2.1063 10444698

[B93] Ohbayashi M. (2020). Inhibition of protein synthesis in M1 of monkeys disrupts performance of sequential movements guided by memory. *Elife* 9:e53038. 10.7554/eLife.53038 32039760 PMC7010406

[B94] Ohbayashi M. Picard N. (2020). Sequential reaching task for the study of motor skills in monkeys. *Bio Protoc.* 10:e3719. 10.21769/BioProtoc.3719 33659383 PMC7853926

[B95] Ohbayashi M. Picard N. Strick P. L. (2016). Inactivation of the dorsal premotor area disrupts internally generated, but not visually guided sequential movements. *J. Neurosci.* 36 1971–1976. 10.1523/JNEUROSCI.2356-15.2016 26865620 PMC4748079

[B96] Orban P. Peigneux P. Lungu O. Albouy G. Breton E. Laberenne F. (2010). The multifaceted nature of the relationship between performance and brain activity in motor sequence learning. *Neuroimage* 49 694–702. 10.1016/j.neuroimage.2009.08.055 19732838

[B97] Passingham R. E. (1988). Premotor cortex and preparation for movement. *Exp. Brain Res.* 70 590–596. 10.1007/BF00247607 3384057

[B98] Pesaran B. Nelson M. J. Andersen R. A. (2008). Free choice activates a decision circuit between frontal and parietal cortex. *Nature* 453 406–409. 10.1038/nature06849 18418380 PMC2728060

[B99] Picard N. Matsuzaka Y. Strick P. L. (2013). Extended practice of a motor skill is associated with reduced metabolic activity in M1. *Nat. Neurosci.* 16 1340–1347. 10.1038/nn.3477 23912947 PMC3757119

[B100] Picard N. Strick P. L. (1997). Activation on the medial wall during remembered sequences of reaching movements in monkeys. *J. Neurophysiol.* 77 2197–2201. 10.1152/jn.1997.77.4.2197 9114266

[B101] Picard N. Strick P. L. (2001). Imaging the premotor areas. *Curr. Opin. Neurobiol.* 11 663–672. 10.1016/s0959-4388(01)00266-5 11741015

[B102] Picard N. Strick P. L. (2003). Activation of the supplementary motor area (SMA) during performance of visually guided movements. *Cereb. Cortex* 13 977–986. 10.1093/cercor/13.9.977 12902397

[B103] Rathelot J. A. Nwankwo A. Strick P. L. (2016). *Origin of Descending Commands from the Cerebral Cortex to Hand Motoneurons in the Rat. Abstract Retrieved from Abstracts in Society of Neuroscience Database Accession No. 534.01.* San Diego, CA: Society for Neuroscience. Available online at: https://www.abstractsonline.com/pp8/index.html#!/4071/

[B104] Reddy P. G. Mattar M. G. Murphy A. C. Wymbs N. F. Grafton S. T. Satterthwaite T. D. (2018). Brain state flexibility accompanies motor-skill acquisition. *Neuroimage* 171 135–147. 10.1016/j.neuroimage.2017.12.093 29309897 PMC5857429

[B105] Roland P. E. Larsen B. Lassen N. A. Skinhoj E. (1980). Supplementary motor area and other cortical areas in organization of voluntary movements in man. *J. Neurophysiol.* 43 118–136. 10.1152/jn.1980.43.1.118 7351547

[B106] Rosenbaum D. A. (2010). *Human Motor Control.* Amsterdam: Elsevier Inc. Publishers.

[B107] Rudy J. W. (2008a). Destroying memories to strengthen them. *Nat. Neurosci.* 11 1241–1242. 10.1038/nn1108-1241 18956008

[B108] Rudy J. W. (2008b). *The Neurobiology of Learning and Memory.* Sunderland, MA: Sinauer Associates, Inc. Publishers.

[B109] Sakai K. Hikosaka O. Miyauchi S. Takino R. Sasaki Y. Putz B. (1998). Transition of brain activation from frontal to parietal areas in visuomotor sequence learning. *J. Neurosci.* 18 1827–1840. 10.1523/jneurosci.18-05-01827.1998 9465007 PMC6792634

[B110] Sampaio-Baptista C. Johansen-Berg H. (2017). White matter plasticity in the adult brain. *Neuron* 96 1239–1251. 10.1016/j.neuron.2017.11.026 29268094 PMC5766826

[B111] Sara S. J. (2000). Strengthening the shaky trace through retrieval. *Nat. Rev. Neurosci.* 1 212–213. 10.1038/35044575 11257910

[B112] Schmidt R. A. Lee T. D. (2011). *Motor Control and Learning: A Behavioral Emphasis.* Champaign, IL: Human Kinetics. Publishers.

[B113] Schwenkreis P. El Tom S. Ragert P. Pleger B. Tegenthoff M. Dinse H. R. (2007). Assessment of sensorimotor cortical representation asymmetries and motor skills in violin players. *Eur. J. Neurosci.* 26 3291–3302. 10.1111/j.1460-9568.2007.05894.x 18028115

[B114] Shibasaki H. Sadato N. Lyshkow H. Yonekura Y. Honda M. Nagamine T. (1993). Both primary motor cortex and supplementary motor area play an important role in complex finger movement. *Brain* 116(Pt 6) 1387–1398. 10.1093/brain/116.6.1387 8293277

[B115] Shima K. Aya K. Mushiake H. Inase M. Aizawa H. Tanji J. (1991). Two movement-related foci in the primate cingulate cortex observed in signal-triggered and self-paced forelimb movements. *J. Neurophysiol.* 65 188–202. 10.1152/jn.1991.65.2.188 2016637

[B116] Shima K. Mushiake H. Saito N. Tanji J. (1996). Role for cells in the presupplementary motor area in updating motor plans. *Proc. Natl. Acad. Sci. U.S.A.* 93 8694–8698. 10.1073/pnas.93.16.8694 8710933 PMC38735

[B117] Shima K. Tanji J. (1998). Both supplementary and presupplementary motor areas are crucial for the temporal organization of multiple movements. *J. Neurophysiol.* 80 3247–3260. 10.1152/jn.1998.80.6.3247 9862919

[B118] Shima K. Tanji J. (2000). Neuronal activity in the supplementary and presupplementary motor areas for temporal organization of multiple movements. *J. Neurophysiol.* 84 2148–2160. 10.1152/jn.2000.84.4.2148 11024102

[B119] Shima K. Tanji J. (2006). Binary-coded monitoring of a behavioral sequence by cells in the pre-supplementary motor area. *J. Neurosci.* 26 2579–2582. 10.1523/JNEUROSCI.4161-05.2006 16510736 PMC6793666

[B120] Takada M. Nambu A. Hatanaka N. Tachibana Y. Miyachi S. Taira M. (2004). Organization of prefrontal outflow toward frontal motor-related areas in macaque monkeys. *Eur. J. Neurosci.* 19 3328–3342. 10.1111/j.0953-816X.2004.03425.x 15217388

[B121] Tanji J. (2001). Sequential organization of multiple movements: involvement of cortical motor areas. *Annu. Rev. Neurosci.* 24 631–651. 10.1146/annurev.neuro.24.1.631 11520914

[B122] Tanji J. Shima K. (1994). Role for supplementary motor area cells in planning several movements ahead. *Nature* 371 413–416. 10.1038/371413a0 8090219

[B123] Tanji J. Shima K. (1996a). Contrast of neuronal activity between the supplemental motor area and other cortical motor areas. *Adv. Neurol.* 70 95–103. 8615234

[B124] Tanji J. Shima K. (1996b). Supplementary motor cortex in organization of movement. *Eur. Neurol.* 36(Suppl. 1) 13–19. 10.1159/000118878 8791016

[B125] Tanji J. Shima K. Mushiake H. (1996). Multiple cortical motor areas and temporal sequencing of movements. *Brain Res. Cogn. Brain Res.* 5 117–122. 10.1016/s0926-6410(96)00047-x 9049077

[B126] Tokuno H. Tanji J. (1993). Input organization of distal and proximal forelimb areas in the monkey primary motor cortex: a retrograde double labeling study. *J. Comp. Neurol.* 333 199–209. 10.1002/cne.903330206 8393892

[B127] Ungerleider L. G. Doyon J. Karni A. (2002). Imaging brain plasticity during motor skill learning. *Neurobiol. Learn. Mem.* 78 553–564. 10.1006/nlme.2002.4091 12559834

[B128] Wang Y. Isoda M. Matsuzaka Y. Shima K. Tanji J. (2005). Prefrontal cortical cells projecting to the supplementary eye field and presupplementary motor area in the monkey. *Neurosci. Res.* 53 1–7. 10.1016/j.neures.2005.05.005 15992955

[B129] Wang Y. Shima K. Sawamura H. Tanji J. (2001). Spatial distribution of cingulate cells projecting to the primary, supplementary, and pre-supplementary motor areas: a retrograde multiple labeling study in the macaque monkey. *Neurosci. Res.* 39 39–49. 10.1016/s0168-0102(00)00198-x 11164252

[B130] Wenger E. Brozzoli C. Lindenberger U. Lovden M. (2017). Expansion and renormalization of human brain structure during skill acquisition. *Trends Cogn. Sci.* 21 930–939. 10.1016/j.tics.2017.09.008 29149999 PMC5697733

[B131] Wiener M. Turkeltaub P. Coslett H. B. (2010). The image of time: a voxel-wise meta-analysis. *Neuroimage* 49 1728–1740. 10.1016/j.neuroimage.2009.09.064 19800975

[B132] Wise S. P. (1985). The primate premotor cortex: past, present, and preparatory. *Annu. Rev. Neurosci.* 8 1–19. 10.1146/annurev.ne.08.030185.000245 3920943

[B133] Wise S. P. Boussaoud D. Johnson P. B. Caminiti R. (1997). Premotor and parietal cortex: corticocortical connectivity and combinatorial computations. *Annu. Rev. Neurosci.* 20 25–42. 10.1146/annurev.neuro.20.1.25 9056706

[B134] Wymbs N. F. Grafton S. T. (2013). Contributions from the left PMd and the SMA during sequence retrieval as determined by depth of training. *Exp. Brain Res.* 224 49–58. 10.1007/s00221-012-3287-1 23283418 PMC3539248

[B135] Xu T. Yu X. Perlik A. J. Tobin W. F. Zweig J. A. Tennant K. (2009). Rapid formation and selective stabilization of synapses for enduring motor memories. *Nature* 462 915–919. 10.1038/nature08389 19946267 PMC2844762

[B136] Yang G. Pan F. Gan W. B. (2009). Stably maintained dendritic spines are associated with lifelong memories. *Nature* 462 920–924. 10.1038/nature08577 19946265 PMC4724802

[B137] Yokoi A. Diedrichsen J. (2019). Neural organization of hierarchical motor sequence representations in the human neocortex. *Neuron* 103 1178–1190.e7. 10.1016/j.neuron.2019.06.017 31345643

[B138] Yu X. Zuo Y. (2011). Spine plasticity in the motor cortex. *Curr. Opin. Neurobiol.* 21 169–174. 10.1016/j.conb.2010.07.010 20728341 PMC2991530

[B139] Zatorre R. J. Fields R. D. Johansen-Berg H. (2012). Plasticity in gray and white: neuroimaging changes in brain structure during learning. *Nat. Neurosci.* 15 528–536. 10.1038/nn.3045 22426254 PMC3660656

